# A successful nursing education promotes newly graduated nurses’ job satisfaction one year after graduation: a cross-sectional multi-country study

**DOI:** 10.1186/s12912-023-01438-y

**Published:** 2023-08-14

**Authors:** Sanna Koskinen, Anna Brugnolli, Pilar Fuster-Linares, Susan Hourican, Natalja Istomina, Helena Leino-Kilpi, Eliisa Löyttyniemi, Jana Nemcová, Gabriele Meyer, Célia Simão De Oliveira, Alvisa Palese, Marília Rua, Leena Salminen, Herdís Sveinsdóttir, Laura Visiers-Jiménez, Renáta Zeleníková, Satu Kajander-Unkuri, on behalf of the ProCompNurse Consortium and the COMPEUnurse Consortium

**Affiliations:** 1 Department of Nursing Science, University of Turku, 20014 Turku, Finland; 2 Azienda Per I Servizi Sanitari Provinciali, University of Verona, 38123 Trento, Italy; 3 Department of Nursing, Universitat Internacional de Catalunya, 08017 Barcelona, Spain; 4 School of Nursing, Psychotherapy and Community Health, Dublin City University, Dublin 9, Ireland; 5 Institute of Health Sciences, Vilnius University, 01513 Vilnius, Lithuania; 6 Turku University Hospital, 20521 Turku, Finland; 7 Department of Biostatistics, University of Turku, 20014 Turku, Finland; 8 Department of Nursing Science, Jessenius Faculty of Medicine in Martin, Comenius University in Bratislava, 03601 Martin, Slovakia; 9 Institute of Health and Nursing Science, Martin Luther University Halle-Wittenberg, 06108 Halle (Saale), Germany; 10 Department of Fundamentals of Nursing, Lisbon School of Nursing—ESEL (Escola Superior de Enfermagem de Lisboa), 1600-096 Lisbon, Portugal; 11 Department of Medicine, Udine University, 33100 Udine, Italy; 12 School of Health Sciences, University of Aveiro, 3810-193 Aveiro, Portugal; 13 Faculty of Nursing, University of Iceland, 07 Reykjavík, Iceland; 14 Department of Health Sciences, School of Nursing and Physiotherapy, Universidad Pontificia de Comillas, San Juan de Dios, Fundación San Juan de Dios, Alberto Aguilera, 23, 28015 Madrid, Spain; 15 Department of Nursing and Midwifery, University of Ostrava, 70103 Ostrava, Czech Republic; 16 Diaconia University of Applied Sciences, 00580 Helsinki, Finland

**Keywords:** Intention to stay, Job satisfaction, Newly graduated nurse, Nursing, Nursing education, Transition

## Abstract

**Background:**

Job satisfaction is a key factor for the successful transition of newly graduated nurses (NGNs) and for retaining NGNs in their workplaces. However, there is limited evidence of the relationship between satisfaction regarding the nursing education program and NGNs’ job satisfaction in the first year after graduation. Therefore, this study aims to examine the association of the nursing education related factors and NGNs’ job satisfaction.

**Methods:**

A cross-sectional study design with the utilization of data collected from the same respondents one year earlier as educational factors was applied. The data were collected from NGNs (*n* = 557) in 10 European countries using an electronic survey between February 2019 and September 2020, and analyzed in detail for four countries (*n* = 417). Job satisfaction was measured with three questions: satisfaction with current job, quality of care in the workplace, and nursing profession. Nursing education related factors were satisfaction with nursing education program, level of study achievements, nursing as the 1st study choice, intention to stay in nursing, and generic nursing competence. The data were analyzed statistically using logistic regression.

**Results:**

Most of the NGNs in the 10 countries were satisfied with their current job (88.3%), the quality of care (86.4%) and nursing profession (83.8%). Finnish, German, Lithuanian and Spanish NGNs’ satisfaction with the nursing education program at graduation was statistically significantly associated with their job satisfaction, i.e., satisfaction with their current job, the quality of care, and the nursing profession. Moreover, NGNs who had fairly often or very often intention to stay in nursing at graduation were more satisfied with their current job, with the quality of care, and with the nursing profession compared with NGNs who had never or fairly seldom intention to stay in nursing at graduation.

**Conclusions:**

Nursing education plays a significant role in NGNs’ job satisfaction one year after graduation, indicating the importance to start career planning already during nursing education. Both nursing education providers and healthcare organizations could plan in close collaboration a transition program for NGNs to ease the transition phase and thus increase the NGNs’ job satisfaction and ultimately the high-quality care of the patients.

## Background

Today, nurses’ job satisfaction is of great interest as the healthcare industry is undoubtedly facing its greatest challenge, the shortage of nurses [1]. Coronavirus disease (hereafter COVID-19) pandemic has caused challenges to health care worldwide [2, 3]: it has increased the need for nurses globally, and the demand will rise further over the next few years [4]. Prior to the pandemic, the World Health Organization estimated the shortage of nurses to be 5.9 million [1]; since then, the situation has exacerbated further as many nurses have died, fallen ill, left their employment or retired prematurely [4]. Increased chronic diseases across populations and nurses’ retirement, where one out of six of the world’s nurses is estimated to retire by the year 2032 [4], worsen the shortage of nurses [5, 6]. New nursing workforce is needed to respond to increasing demands and to secure safe and high-quality patient care in the future [1, 7]; for organizations, retaining newly graduated nurses (hereafter NGNs) is thus vital. However, recent studies have indicated that NGNs planned to leave their job during the first year of employment [8–11]. This trend may worsen the current nurse shortage crisis and pose a threat to high-quality patient care and patient safety. Job satisfaction has been found to be a key factor for successful transition of NGNs to the world of work [12–14] and for retaining NGNs in their workplaces [15–17].

Nurses’ job satisfaction can be defined as “nurses’ positive feeling response to the work conditions that meet his or her desired needs as the result of their evaluation of the value or equity in their work” ([14], p. 87). Nurses’ job satisfaction is essential because of its association with high patient care quality [12, 17, 18], which is the final goal of every healthcare organization. Job satisfaction has been linked to nurses’ autonomy and to finding work meaningful [19]. There is also a positive association between job satisfaction and nurses’ psychological well-being [18] and job performance [14].

Among NGNs, job satisfaction is associated with satisfaction with career [20], organizational commitment [21, 22], work-life balance [12], job-related stress [23], and some working environment factors [24–26], especially staffing adequacy [12, 22, 27] and structural empowerment [20, 27]. Job satisfaction has been reported to increase over time by one year of work experience [23, 28]. This first year of employment, also known as “transition phase”, is crucial and considered a special phase for NGNs. When entering nursing practice as professionals, NGNs are happy and excited about their new jobs and open for learning [29]. During this phase they gain experiences which influence their commitment to the profession and career planning [22].

Facilitating a successful transition and the beginning of a nursing career should start already during nursing education [30, 31]. In available studies, most of the NGNs had issues with self-trust in their professional practice [32–34] and knowledge deficits [35], even though their self-assessed competence at graduation was at good level [36]. The influence of a successful final clinical practicum has been found to facilitate the practice readiness of graduating nursing students [37], to ease the transition phase [30, 38], and to promote career retention at graduation [39]. However, there is limited evidence of the relationship between satisfaction with the nursing education program and NGNs’ job satisfaction in the first year after graduation. Kenny et al. [40] and Ulupinar and Aydogan [10] have found that satisfaction with how nursing education prepared NGNs for nursing was associated with satisfaction with job-related factors. To date, there are no studies that have explored the association between the educational factors prevailing at the time of graduation and later job satisfaction.

## Methods

### Aim

This study aimed to examine the association of the nursing education related factors and NGNs’ job satisfaction. The research question was:

1. What nursing education related factors were associated with newly graduated nurses’ job satisfaction one year after graduation?

### Design

A cross-sectional study design with the utilization of data collected from the same respondents one year earlier as educational factors was applied. This study is an independent sub-study of two separate European research projects – Competence of Nursing Students in Europe (COMPEUnurse) and Professional Competence in Nursing (ProCompNurse). Both projects focus on nursing students’ competence and possible factors associated with it at the time of graduation and in the first years of the career. Nursing students from 10 European countries [Czech Republic (CZ), Finland (FI), Germany (DE), Iceland (IS), Ireland (IE), Italy (IT), Lithuania (LT), Portugal (PT), Slovakia (SK), and Spain (ES)] have joined in these study projects by responding to different research instruments at graduation (T1 pre-graduation data) and one year after graduation (T2 post-graduation data). In these countries, nursing education follows the European Union directives (2005/36/EC, 2013/55/EU) and is offered at universities (CZ, DE, IS, IE, IT, LT, SK, ES), universities of applied sciences (FI), polytechnic institutes (PT) or colleges (CZ, DE, LT, SK) at higher educational level (Kajander-Unkuri et al., 2021). This study focuses on NGNs’ job satisfaction one year after graduation (T2) and the associated educational factors (T1). The study reporting was complied with the “Strengthening the Reporting of Observational studies in Epidemiology” (STROBE) guidelines [41].

### Participants and procedure

The study population consisted of NGNs after one year of work experience from the ten above-mentioned European countries which are located geographically in different parts of Europe. The convenience sample was based on the first phase of the study projects, which also included a convenience sample of graduating nursing students from the eligible countries. In this second phase, respondents who had given their contact details in the first phase of the study projects, i.e., newly graduated nurses after one year of work experience (*N* = 2,792), were examined. A total of 557 NGNs responded to the survey. In this article, the results of NGNs from four countries, namely Finland, Germany, Lithuania, and Spain (*n* = 417), are presented in more detail. The results of NGNs from six other countries (*n* = 140) are only described descriptively due to the small number of respondents in these countries.

The data of this study was collected between February 2019 and September 2020. The data were collected by using an electronic questionnaire (COMPEUnurse: Webropol; ProCompNurse: otherwise, REDCap [42], but in Germany, SoSci Survey software) with a national language version. At the onset of the projects, the questionnaires were piloted in each country to ensure their feasibility and understandability [36]. The projects’ contact persons sent the survey to the email addresses provided by the NGNs at T1 at the time when they were graduating nursing students. To increase the response rate, two reminders were sent [43, 44]. The surveys were coded with an anonymized identification code to enable statistical analyses.

Both projects respected the ethical principles of the Declaration of Helsinki [45] and the responsible conduct of research [46]. At T1, permission for using and translating the research instruments was obtained from the copyright holders and the research permissions were granted by all participating educational institutions according to national standards. Confidentiality and voluntarily were guaranteed during the recruitment process. All participants received an information letter about the study which contained sufficient details to enable them to make an informed decision on participating in the study. Participants signed an informed consent when they agreed to participate in the study at T1 and provided their email address for T2 data collection. Consent was requested again at T2.

### Measures

Job satisfaction was measured with three questions: satisfaction with current job, quality of care in the workplace, and nursing profession using a 4-point Likert scale (from 1 = fully disagree to 4 = fully agree). These three questions have been used successfully earlier in a study surveying likewise NGNs [25].

In addition to demographic information (age, gender, country), nursing education related factors (data at graduation point, T1) were used as background factors: (1) satisfaction with nursing education program (very unsatisfied‒very satisfied), (2) level of study achievements (very poor‒excellent), (3) nursing as the 1st study choice (yes/no), (4) intention to stay in nursing (never‒very often), and (5) generic nursing competence (very low‒very high) evaluated with the Nurse Competence Scale (NCS) [47]. The NCS was used as a background factor dividing the NGNs into three groups on the basis of their total Visual Analogue Scale (VAS) score at graduation (rather good: VAS mean < 50, good: VAS > 50–75, and very good: VAS > 75–100). The validity and reliability of the NCS has been demonstrated in numerous international studies in different countries and nursing contexts [36, 47, 48]. In this study, the internal consistency (Cronbach's alpha) for the total sample was 0.98. Additionally, for the four countries included in the logistic regression analysis, where the number of respondents was sufficient, the internal consistency varied from 0.96 (Germany) to 0.98 (Finland, Lithuania, and Spain). These values align with earlier studies [48].

### Statistical analysis

Descriptive statistics were used for the 10-country sample. Continuous and normally distributed data were summarized using mean and standard deviation (SD) and categorical variables with counts and percentages. Due to relatively small number of respondents in several countries we decided to include countries into further statistical analysis when the number of respondents per country exceeded 50. In addition, due to the small number of respondents answering the end options of the 4-point Likert scale (fully disagree and fully agree), the options ‘fully disagree’ and ‘disagree’ were merged as ‘disagree’ as well as ‘agree’ and ‘fully agree’ were merged as ‘agree’ in the data analysis.

Association between job satisfaction variables and demographic factors (age and gender) as well as educational factors (satisfaction with nursing education program, level of study achievements, nursing as the 1st study choice, intention to stay in nursing, and generic nursing competence) was studied with logistic regression including country (Finland, Germany, Lithuania, and Spain based on their sample size) in each model, and other factors were added one at a time due to collinearity issue of background variables. If the background factor was statistically significant, contrasts were created to study which category differed from the others.

In all analyses, participants with the incomplete background or educational variables were automatically removed from the statistical analyses. In the statistical analyses, a significance level of 0.05 (two-tailed) was used. The data analysis was performed using SAS software, Version 9.4 of the SAS System for Windows (SAS Institute Inc., Cary, NC, USA).

## Results

### Characteristics of the participants

As for the overall 10-country sample, the NGNs (*n* = 557) were mostly women (86.9%) and their mean age was 27.5 years (SD 7.2, range 21–61 years). Over four-fifths (85.0%) were satisfied or very satisfied with their nursing education program as a whole, and over three quarters (78.0%) had nursing as their first study choice. Over half of the NGNs (61.7%) had assessed their generic nursing competence to be at good level (VAS > 50–75) at graduation. Most of the NGNs were satisfied with their current job, with the quality of care in their workplaces, and with the nursing profession (88.3%, 86.4% and 83.8%, respectively). Regarding individual countries, Icelandic NGNs (100%) were the most satisfied with their current job, while Czech NGNs (80.0%) were the least satisfied. Spanish NGNs (92.2%) were the most satisfied with the quality of care in the workplace, whereas Slovakian NGNs (75.0%) were the least satisfied. Slovakian NGNs (93.7%) were the most satisfied with the nursing profession, whereas Portuguese NGNs (53.1%) were the least satisfied (Table 1).

**Table 1:** Characteristics of the 10-country sample (*n* = 557)

Characteristics	Czech Republic (*n* = 15)	Finland (*n* = 233)	Germany (*n* = 63)	Iceland (*n* = 22)	Ireland (*n* = 22)	Italy (*n* = 33)	Lithuania (*n* = 57)	Portugal (*n* = 32)	Slovakia (*n* = 16)	Spain (*n* = 64)	Total (*n* = 551–557)
**Age (years)**^a^**, T2,** Mean (SD)	27.7 (8.7)	30.2 (7.6)	25.0 (5.5)	28.5 (5.2)	26.0 (6.3)	24.0 (2.2)	26.6 (7.4)	22.9 (2.0)	24.5 (3.4)	26.2 (7.4)	27.5 (7.2)
Min–max	23–49	22–57	21–49	23–42	21–43	22–32	21–61	21–31	22–36	21–57	21–61
	**n (%)**	**n (%)**	**n (%)**	**n (%)**	**n (%)**	**n (%)**	**n (%)**	**n (%)**	**n (%)**	**n (%)**	**n (%)**
**Gender**^a^**, T2**											
Female	14 (93.3)	202 (87.4)	44 (74.6)	21 (95.4)	18 (81.8)	27 (81.8)	55 (96.5)	26 (81.2)	15 (93.7)	57 (89.1)	479 (86.9)
Male	1 (6.7)	29 (12.6)	15 (25.4)	1 (4.6)	4 (18.2)	6 (18.2)	2 (3.5)	6 (18.8)	1 (6.3)	7 (10.9)	72 (13.1)
**Satisfaction with nursing education**^a^**, T1**											
Satisfied / very satisfied	10 (66.7)	178 (80.9)	47 (79.7)	20 (100)	16 (88.9)	30 (90.9)	46 (83.6)	27 (90.0)	16 (100)	56 (94.9)	446 (85.0)
Unsatisfied / very unsatisfied	5 (33.3)	42 (19.1)	12 (20.3)	0	2 (11.1)	3 (9.1)	9 (16.4)	3 (10.0)	0	3 (5.1)	79 (15.0)
**Level of study achievements**^a^**, T1**											
Excellent	0	26 (11.8)	8 (13.6)	4 (20.0)	4 (22.2)	5 (15.2)	8 (14.6)	9 (28.1)	2 (12.5)	11 (18.6)	77 (14.6)
Good	13 (86.7)	188 (85.5)	51 (86.4)	16 (80.0)	14 (77.8)	28 (84.8)	44 (80.0)	23 (71.9)	14 (87.5)	45 (76.3)	436 (82.7)
Very poor / poor	2 (13.3)	6 (2.7)	0	0	0	0	3 (5.4)	0	0	3 (5.1)	14 (2.7)
**Nursing as the 1**^**st**^** study choice**^a^ ***(Yes),* T1**	11 (78.6)	205 (88.7)	33 (55.9)	11 (50.0)	19 (86.4)	30 (90.9)	35 (61.4)	28 (87.5)	13 (81.3)	44 (68.8)	429 (78.0)
**Nurse competence (NCS)**^a^**, T1**											
VAS < 50	0	40 (17.7)	5 (8.3)	1 (5.0)	3 (13.6)	0	27 (48.2)	3 (9.4)	5 (31.3)	7 (11.5)	91 (16.9)
VAS > 50–75	9 (60.0)	140 (62.0)	43 (71.7)	13 (65.0)	15 (68.2)	24 (75.0)	25 (44.6)	20 (62.5)	8 (50.0)	36 (59.0)	333 (61.7)
VAS > 75–100	6 (40.0)	46 (20.3)	12 (20.0)	6 (30.0)	4 (18.2)	8 (25.0)	4 (7.2)	9 (28.1)	3 (18.7)	18 (29.5)	116 (21.5)
**Intention to stay in nursing**^a^**, T1**											
Fairly often / very often	12 (80.0)	171 (73.4)	38 (64.4)	16 (72.7)	15 (68.2)	31 (93.9)	43 (75.4)	23 (71.9)	15 (93.8)	59 (92.2)	423 (76.5)
Never / fairly seldom	3 (20.0)	62 (26.6)	21 (35.6)	6 (27.3)	7 (31.8)	2 (6.1)	14 (24.6)	9 (28.1)	1 (6.2)	5 (7.8)	222 (23.5)
**Satisfied with current job, T2**											
Agree	12 (80.0)	207 (88.8)	55 (87.3)	22 (100)	20 (90.9)	28 (84.8)	51 (89.5)	27 (84.4)	14 (87.5)	56 (87.5)	492 (88.3)
Disagree	3 (20.0)	26 (11.2)	8 (12.7)	0	2 (9.1)	5 (15.2)	6 (10.5)	5 (15.6)	2 (12.5)	8 (12.5)	65 (11.7)
**Satisfied with the quality of care in the workplace, T2**											
Agree	12 (80.0)	201 (86.3)	52 (82.5)	20 (90.9)	20 (90.9)	26 (78.8)	50 (87.7)	29 (90.6)	12 (75.0)	59 (92.2)	481 (86.4)
Disagree	3 (20.0)	32 (13.7)	11 (17.5)	2 (9.1)	2 (9.1)	7 (21.2)	7 (12.3)	3 (9.4)	4 (25.0)	5 (7.8)	76 (13.6)
**Satisfied with nursing profession, T2**											
Agree	13 (86.7)	203 (87.1)	44 (69.8)	20 (90.9)	19 (86.4)	28 (84.8)	51 (89.5)	17 (53.1)	15 (93.7)	57 (89.1)	467 (83.8)
Disagree	2 (13.3)	30 (12.9)	19 (30.2)	2 (9.1)	3 (13.6)	5 (15.2)	6 (10.5)	15 (46.9)	1 (6.3)	7 (10.9)	90 (16.2)

*Abbreviations*: *SD* Standard deviation, *VAS* Visual Analogue Scale, *NCS* Nurse Competence Scale, *T1* Pre-graduation data, *T2* Post-graduation data

^a^Variable includes variety of missing values

As for the 4-country sample (Finland, Germany, Lithuania, Spain), the NGNs (*n* = 417) were mostly women (87.1%) and their mean age was 28.3 years (SD 7.6, range 21–61 years). Over four-fifths (83.2%) were satisfied or very satisfied with their nursing education program as a whole, and over three quarters (77.1%) had nursing as their first study choice. Over half of the NGNs (60.5%) had assessed their generic nursing competence to be at good level (VAS > 50–75) at graduation. Most of the NGNs were satisfied with their current job, with the quality of care in their workplaces, and with the nursing profession (88.5%, 86.8% and 85.1%, respectively) (Table 1). Next, the results only from these four countries will be reported.

### The association between the nursing education related factors and the NGNs’ job satisfaction

The logistic regression including only those countries with more than 50 participants (Germany, Finland, Lithuania, and Spain) indicated a high association between the NGNs’ satisfaction with the nursing education program at graduation and satisfaction with the current job (*p* = 0.0082), with the quality of care in the workplace (*p* = 0.042), and with the nursing profession (*p* < 0.0001). In addition, NGNs who rated their study achievements at graduation as good were more satisfied with the nursing profession than NGNs who rated their study achievements as very poor or poor (*p* = 0.021). Moreover, NGNs who had fairly often or very often intention to stay in nursing at graduation were more satisfied with their current job (*p* < 0.0001), with the quality of care in the workplace (*p* = 0.0003), and with the nursing profession (*p* < 0.0001) compared with NGNs who had never or fairly seldom intention to stay in nursing at graduation (Table 2).

**Table 2:** The association of background factors with job satisfaction analysed with logistic regression including country (*n* = 417)

Background factor	Satisfaction with current job *p*-value	Satisfaction with the quality of care *p*-value	Satisfaction with nursing profession *p*-value
**Age, T2**	0.52	0.85	0.012*
**Gender, T2**	0.86	0.081	0.78
**Country, T2**	0.97	0.45	0.0046*
Finland vs Germany			0.0015*
Finland vs Lithuania			0.63
Finland vs Spain			0.68
Germany vs Lithuania			0.011*
Germany vs Spain			0.0096*
Lithuania vs Spain			0.94
**Satisfaction with nursing education, T1**	0.0082*	0.042*	< 0.0001*
Very unsatisfied vs unsatisfied	0.026*	0.009*	0.36
Very unsatisfied vs satisfied	0.0018*	0.035*	0.0087*
Very unsatisfied vs very satisfied	0.0032*	0.035*	0.0055*
Unsatisfied vs satisfied	0.13	0.051	0.0001*
Unsatisfied vs very satisfied	0.16	0.051	0.0035*
Satisfied vs very satisfied	0.62	0.011*	0.32
**Level of study achievements, T1**	0.097	0.93	0.019*
Very poor/poor vs good			0.021*
Very poor/poor vs excellent			0.27
Good vs excellent			0.058
**Nursing as the 1**^**st**^** study choice *(Yes),* T1**	0.65	0.95	0.36
**Nurse competence (NCS), T1**			
Total	0.30	0.44	0.13
Class	0.13	0.24	0.36
**Intention to stay in nursing, T1**	< 0.0001*	0.0003*	< 0.0001*
Very often vs fairly often	0.039*	0.41	0.36
Very often vs fairly seldom	< 0.0001*	0.012*	< 0.0001*
Very often vs never	< 0.0001*	0.0002*	< 0.0001*
Fairly often vs fairly seldom	0.0030*	0.039*	< 0.0001*
Fairly often vs never	0.0006*	0.0005*	< 0.0001*
Fairly seldom vs never	0.53	0.069	0.062

*Abbreviation*: *NCS* Nurse Competence Scale, *T1* Pre-graduation data, *T2* Post-graduation data

*Statistically significant *p*-value

### The association between the demographic factors and the NGNs’ job satisfaction

The logistic regression analysis also revealed that older NGNs were more satisfied with the nursing profession compared with younger NGNs (*p* = 0.012). The mean age of NGNs who were the most satisfied with the nursing profession was 28.8 years (SD 7.9, range 21–61 years) while the mean age of NGNs who were the least satisfied was 25.7 years (SD 4.4, range 22–41 years). Satisfaction with nursing profession differed between the countries. NGNs from Finland, Lithuania and Spain were more satisfied with the nursing profession compared with German NGNs (*p* = 0.0015, *p* = 0.011, *p* = 0.0096, respectively) (Table 2).

## Discussion

This study aimed to examine the association of the nursing education related factors and NGNs’ job satisfaction. Although not all studied countries could be analyzed for this association, they were included in the article with the intention of providing a broader perspective and context to the study, shedding light on the sample of early career nurses across Europe and their job satisfaction. This approach may inspire further comparisons and investigations in this field [49]. Additionally, by including all countries, the study and its results are reported transparently, completely, and honestly [46, 50].

The main finding is that satisfaction with the nursing education program was positively associated with NGNs’ job satisfaction, i.e., satisfaction with their current job, with the quality of care in their workplaces, and with the nursing profession. The finding is new as there are no quantitative studies relating satisfaction with the nursing education program to NGNs’ job satisfaction, where satisfaction with nursing education is measured at graduation and job satisfaction one year after graduation suggesting that educational factors can carry until the end of the transition phase. However, quite similarly, Ulupinar and Aydogan [10] found that NGNs who considered their nursing education to be sufficient adapted easily to nursing and their working units and had higher levels of professional satisfaction. Furthermore, Kenny et al. [40] found that satisfaction with how the nursing education prepared one for work as a nurse was related to satisfaction with the physical work environment, the current work hours and salary. However, contrary to the present study, in both studies all measurements were conducted while NGNs were already working as professionals meaning that NGNs evaluated their nursing education retrospectively together with the current work situation which might have biased both evaluations. In the present study, the evaluations of the nursing education and job satisfaction were recorded independently from the same participants assumingly decreasing confounding of the evaluations. With similar design among newly graduated social workers, Hussein et al. [51] found that newly graduated social workers’ job satisfaction was predicted by the extent to which they felt that their degree program had prepared them for their current job.

In addition, good study achievements during nursing education associated positively with NGNs’ satisfaction with the nursing profession, which supports the relationship between nursing education and NGN’s job satisfaction. It might be that nursing students assessing their study achievements as good at graduation had gained a more positive professional identity [52] and were therefore more satisfied with the nursing profession. Ulupinar and Aydogan [10] found that NGNs who considered themselves competent in terms of professional knowledge and skills did not have difficulties adapting to their profession and their levels of satisfaction were higher. In our study, there was no statistically significant association between self-assessed competence at graduation and job satisfaction. In the future, the association could be measured at the same time point.

Intention to stay in nursing profession was associated with the NGNs’ job satisfaction, which is consistent with earlier studies [9, 15–17]. However, it is notable that in our study, NGNs’ intention to stay in nursing was measured at graduation, i.e., one year before measuring the job satisfaction. This highlights the importance of nursing education in preparing the nursing students for the reality of nurse’s job and the nursing profession. In previous studies, successful final clinical practicum has been found to facilitate practice readiness [37] and the competence of nursing students at graduation [39]. Moreover, career development has been found to be positively associated with the intention to stay in nursing [53], and nursing students with access to a career planning and development program during their nursing education had higher perceived career resilience [54]. During nursing education, it is important to identify the students who have thoughts of not staying in nursing after graduation. Every effort should be made to ease the transition phase and prevent these new nurses from leaving the profession. Therefore, during the nursing education, it is essential to start the career planning of nursing students from graduation onwards and this connection requires investigation in the future.

Older NGNs were more satisfied with the nursing profession after one year of employment compared with younger NGNs. One reason for this might be due to generational characteristics and resulting in variations what is valued in work, and whether the satisfaction items covered in the present study grasped these aspects for all respondents [55, 56]. Overall, NGNs belonging to millennials (also referred as generation Y, born 1981–2000) have reported lower job satisfaction compared with baby boomers (born 1946–1964) and generation X (born 1963–1980) [53, 54] and it has been reported that COVID-19 pandemic affected the youngest nurses the most [57]. In the coming years, more millennials will be joining the workforce. It is important for nurse managers to anticipate intergenerational characteristics among NGNs and provide supportive working environments that recognize these characteristics. For nursing education, anticipating the generational characteristics is also important as generation Z has entered higher education [58]. However, this result call for more investigation and validation.

The results of this study have important implications for nurse educators, nursing education providers, nurse managers, and healthcare organizations. The implication for nurse educators is to identify the students who have thoughts of not staying in nursing after graduation. Every effort should be made to prevent these students from putting their thoughts into action when they graduate. Understanding the possible intergenerational characteristics and the different needs of NGNs based on generations is also important for nurse educators when they choose their teaching methods for different courses. Nursing education providers could offer the career planning from graduation onwards. This might improve the vocational identity, too. Students’ satisfaction of their nursing education could be assessed systematically, as it is part of the quality of education. Nurses’ job satisfaction has an impact on high-quality patient outcomes. Therefore, nurse managers could consider the professional expectations of NGNs to increase their job satisfaction. For managers, it is important to understand the intergenerational characteristics and the different needs of NGNs based on generations to support NGNs’ professional transition from education to practice. In addition, both nursing education providers and healthcare organizations could plan in close collaboration a transition program for newly graduated nurses to ease the transition phase and thus increase the NGNs’ job satisfaction and ultimately the high-quality care of the patients.

The limitations of this study have to do with the sample, which was relatively small in some countries preventing their inclusion in the logistic regression analysis. This might have influenced the statistical associations found. The data collection ended in some countries when the first wave of COVID-19 had hit Europe and therefore, responding to the online questionnaire might not have been the NGNs’ first priority. In addition to a convenience sample, there might also be a selection bias as dissatisfied NGNs may not have responded. Only cautious conclusions can be made, and the generalizability of the results is limited due to the low representativeness of the sample especially for Germany and Spain in terms of country size. A multi-country study with a larger sample from different European countries would be important for future consideration. It is also notable that the researchers could not control the possible variables that NGNs had been facing in their workplaces during the transition, particularly during difficult times such as those related to the pandemic. Several variables of the study were surveyed with single-items which can raise validity and reliability concerns although they are adequate for concrete and unidimensional constructs [59]. As an example, the satisfaction with nursing education at graduation was measured with one question. This measure might have lacked sensitivity for this purpose even though statistically significant associations were found and thus further research, preferably qualitative, to gain deeper understanding about satisfaction with nursing education is needed in the future. However, this result shows the importance of satisfaction with nursing education for a fundamental thing such as job satisfaction.

## Conclusions

The results of this study offer a new perspective into NGNs’ job satisfaction, adding new knowledge to the existing body of evidence. The current study highlights the importance of successful nursing education in NGNs’ job satisfaction one year after graduation. In addition, intention to stay in nursing at graduation was associated with job satisfaction. Hence, nursing education has a significant role in NGNs’ job satisfaction one year after graduation and career planning should start already during nursing studies. The results also show that NGNs’ age was associated with their job satisfaction. This might refer to intergenerational characteristics. For nurse educators and managers, it is important to understand these characteristics and the different needs of NGNs based on generations to support NGNs’ professional transition from education to practice.

## Data Availability

The data that support the findings of this study are not openly available due to reasons of privacy and are available from the corresponding author upon reasonable request. Data are located in controlled access data storage at the University of Turku.

## Abbreviations

COVID-19: Coronavirus disease
NCS: Nurse Competence Scale
NGN: Newly graduated nurse
SAS: Statistical Analysis Software
SD: Standard deviation
T1: Pre-graduation data
T2: Post-graduation data
VAS: Visual Analogue Scale
